# In Vitro/In Vivo Correlation of Two Extended-Release Cilostazol Formulations

**DOI:** 10.3390/ph17060787

**Published:** 2024-06-16

**Authors:** Kyoung Ah Min, Na Young Kim, Min Jeong Jin, Doyeon Kim, Yoonseo Ma, Sandeep Karna, Young-Joon Park

**Affiliations:** 1 College of Pharmacy and Inje Institute of Pharmaceutical Sciences and Research, Inje University, 197 Injero, Gimhae 50834, Gyeongnam, Republic of Korea; minkahh@inje.ac.kr (K.A.M.); ggack0103@naver.com (D.K.); 2 College of Pharmacy, Ajou University, 206 Worldcup-ro, Yeongtong-gu, Suwon 16499, Gyeonggi-do, Republic of Korea; kny1998@ajou.ac.kr (N.Y.K.); jmjpypy@ajou.ac.kr (M.J.J.); mayoonseo@naver.com (Y.M.); skarna@gmail.com (S.K.); 3 Research Center, IMD Pharm Inc., 17 Daehak 4-ro, Yeongtong-gu, Suwon 16226, Gyeonggi-do, Republic of Korea

**Keywords:** capsule, cilostazol, release, in vitro, in vivo, pharmacokinetics, two-period crossover design

## Abstract

This study aims to evaluate and determine the correlation between in vitro release and in vivo pharmacokinetics of two extended-release dosage forms of Cilostazol. In vitro release profiles for two dosage forms, tablet and capsule, were analyzed under physiologically mimicked medium conditions using the paddle and basket USP release apparatus. A single-dose, two-period crossover study design in beagle dogs was applied for the pharmacokinetic study. The fed and fast effects were considered for evaluation. Pseudo gastric release medium transfer setup study from pH 1.2 to pH 6.8 (+0.5% SLS) and pH 1.2 to pH 6.8 (+1.0% SLS) demonstrated that Pletaal^®^ SR 200 mg capsules have higher drug release rates than Cilostan^®^ CR 200 mg tablets. Similarly, in vivo study showed Cilostazol concentration in plasma and AUC was lower under the fast state than the fed state. The ratio of least squared geometric mean values, Cmax, AUC_0-t_, and AUC_0-inf_ of Cilostazol were 2.53-fold, 2.89-fold, and 2.87-fold higher for Pletaal^®^ SR 200 mg capsules compared with Cilostan^®^ CR 200 mg tablets, respectively. Correlation of in vitro/in vivo data indicated that Pletal^®^ SR 200 mg capsules have better release and pharmacodynamic effect than Cilostan^®^ CR 200 mg tablets.

## 1. Introduction

Cilostazol is a synthetic vasodilator with an antiplatelet effect and has been used for treating intermittent claudication caused by peripheral arterial disease [1,2,3]. A Pletaal^®^ tablet (Otsuka Pharmaceutical Co., Ltd., Tokyo, Japan), containing Cilostazol, was approved to treat ischemic symptoms of patients with peripheral arterial occlusion in several countries including Japan [4]. Side effects like tachycardia, headache, or dizziness have been reported for the Cilostazol immediate-release drug; therefore, some patients who encounter sudden side effects choose to stop taking medications [5,6]. Thus, sustained-release formulations of Cilostazol with once-daily dosing regimens have been developed for improved patient compliance and therapeutic efficacy [7,8,9,10,11].

In the Republic of Korea, the Pletaal^®^ SR 200 mg capsule (Korea Otsuka Pharmaceutical Co., Ltd., Seoul, Republic of Korea) has been widely used in clinics as a sustained-release formulation of Cilostazol since its approval on 19 April 2011. The Cilostan^®^ CR (Korea United Pharmaceutical Co., Ltd., Seoul, Republic of Korea) was approved as a controlled-release tablet on 28 February 2013. The Pletaal^®^ SR 200 mg formulation is made with a dispersion of starch and Cilostazol for fostering gastrointestinal (GI) resistant properties. Starch mixtures have been widely used in various extended-release formulations because of their resistant activity to digestive enzymes or harsh conditions in the GI tract [12,13,14], and played an important role in maintaining sustained drug release [15,16,17]. While the Cilostan^®^ CR 200 mg tablet contains Cilostazol and matrix-type polymers with pH-dependent drug release [18].

Cilostazol’s metabolism study showed that cytochrome P450 enzymes convert Cilostazol to pharmacologically active metabolites OPC-13015 and OPC-13213 [19]. Firstly, Cilostazol is metabolized to OPC-13326 by Cytochrome P450 3A4 followed by OPC-13015. Similarly, Cilostazol is metabolized to OPC-32217 by Cytochrome P450 3A5 followed by OPC-13213 [19,20]. OPC-13015 has a 3-fold potency of inhibiting platelet aggregation, compared with cilostazol, whereas OPC-13213 is 3-fold less potent than Cilostazol [20]. Since OPC-13015 is a highly active molecule compared with the parent drug, Cilostazol, pharmacokinetic (PK) profiles of OPC-13015 have also been analyzed in several studies [9,21]. Recently, Shin et al.’s clinical study reported a comparison in PK profiles between Pletaal^®^ SR 200 mg capsules and Cilostan^®^ CR 200 mg tablets after once-daily oral administration in human volunteers [22]. A noteworthy conclusion arrived at by this study was that both brands were not bioequivalent in a fast condition. Still, there is a need to investigate more in vitro release profiles and in vivo pharmacokinetic studies between Pletaal^®^ SR 200 mg capsules and Cilostan^®^ CR 200 mg tablets in preclinical studies. In this study, we extend our research to determine the correlation between in vitro release and in vivo pharmacokinetics of two extended-release dosage forms (tablet and capsule) of Cilostazol.

## 2. Results

### 2.1. In Vitro Drug Release Profiling of Extended-Release Cilostazol Formulations in Different Medium Conditions

**Figure 1:**
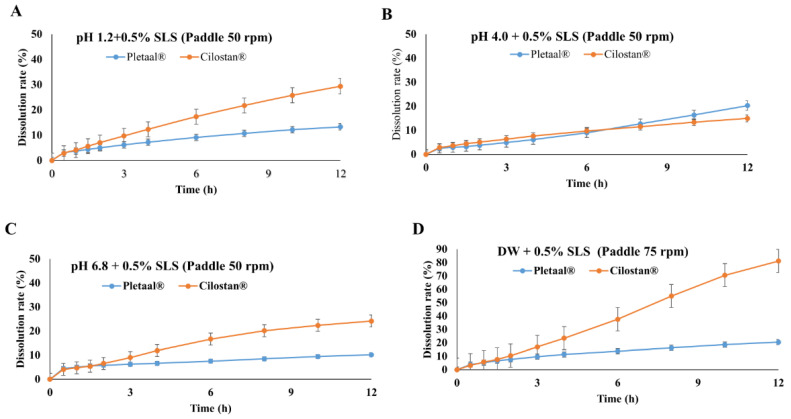
In vitro release profiles of Pletaal^®^ SR 200 mg capsules and Cilostan^®^ CR 200 mg tablets at a paddle speed of 50 rpm. (**A**) Release medium pH 1.2 containing 0.5% SLS (paddle 50 rpm), (**B**) release medium pH 4.0 containing 0.5% SLS (paddle 50 rpm), (**C**) release medium pH 6.8 containing 0.5% SLS (paddle 50 rpm), and (**D**) release medium containing distilled water with 0.5% SLS (paddle 75 rpm).

Three different batches of Cilostan^®^ CR 200 mg tablets (6125105, 6125106, and 6125113) and Pletaal^®^ SR 200 mg capsules (PC215008B, PC215009B, and PC215010B) were assessed for in vitro drug release kinetics in the physiologically mimicked medium, respectively. Firstly, the release medium containing 0.5% SLS (pH 1.2), Cilostan^®^ CR 200 mg tablets, and Pletaal^®^ SR 200 mg capsules showed very low release, lower than 30% (Figure 1A). Pletaal^®^ SR 200 mg capsules were dissolved up to 14% at maximum, whereas Cilostan^®^ CR 200 mg tablets were dissolved up to 30% in the medium, exhibiting more than 2-fold higher release than Pletaal^®^ SR 200 mg capsules. Next, the release medium with pH 4.0, containing 0.5% SLS, Pletaal^®^ SR 200 mg capsules, and Cilostan^®^ CR 200 mg tablets showed very low release profiles, lower than 21% (Figure 1B); however, Pletaal^®^ SR 200 mg capsules showed higher drug release. In the release medium with 0.5% SLS at pH 6.8, Pletaal^®^ SR 200 mg capsules exhibited a release of around 10% up to 12 h (Figure 1C). In the same release medium, conditioned Cilostan^®^ CR 200 mg tablets showed up to 24% drug release which is around 2.4 times higher than Pletaal^®^ SR 200 mg capsules. In the release medium of distilled water containing 0.5% SLS, Cilostan^®^ CR 200 mg tablets showed up to 81% of drug release, while Pletaal^®^ SR 200 mg capsules exhibited only up to 21% drug release (Figure 1D).

**Figure 2:**
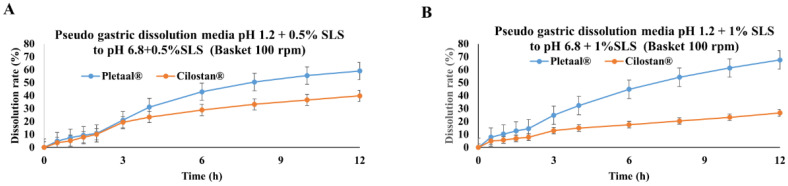
In vitro release profiles of Pletaal^®^ SR 200 mg capsules and Cilostan^®^ CR 200 mg tablets with basket method at 100 rpm. (**A**) Pseudo gastric release medium transfer set up from pH 1.2 to pH 6.8 (+0.5% SLS), and (**B**) Pseudo gastric release medium transfer set up from pH 1.2 to pH 6.8 (+1.0% SLS).

Furthermore, a medium transfer study with pH changes was performed for both formulations, which is known as pseudo gastric release media. Pletaal^®^ SR 200 mg capsules vs. Cilostan^®^ CR 200 mg tablets showed drastic differences in release profile. Higher release rates were exhibited for Pletaal^®^ SR 200 mg capsules than Cilostan^®^ CR 200 mg tablets in the pH transfer setup (Figure 2A,B). Release medium with 0.5% SLS at pH changes from 1.2 to 6.8 (basket 100 rpm), Pletaal^®^ SR 200 mg capsules reached a higher release level of 60%, whereas Cilostan^®^ CR 200 mg tablets showed around 40% release (Figure 2A). Similarly, release medium with 1% SLS at pH changes from 1.2 to 6.8 (basket 100 rpm), Pletaal^®^ SR 200 mg capsules reached a higher release level up to 68%, whereas Cilostan^®^ CR 200 mg tablets showed around 27% release (Figure 2A). The surfactant content is increased in the release medium (from 0.5% to 1% SLS), and the release rate of Pletaal^®^ SR 200 mg capsules was increased overall; at the same time, Cilostan^®^ CR 200 mg tablet drug release decreased. SLS is commonly used as a surfactant to improve the release profile of poorly water-soluble drugs like Cilostazol, since Pletaal^®^ SR 200 mg capsules do not have SLS in the formulation. When we increased the SLS content in the release medium, the drug release profile increased. Similarly, Cilostan^®^ CR 200 tablets already have SLS in the formulation, and when SLS content increases in the release medium, it can lead to increased viscosity, and micellar saturation, which has a negative impact on the release profile.

**Figure 3:**
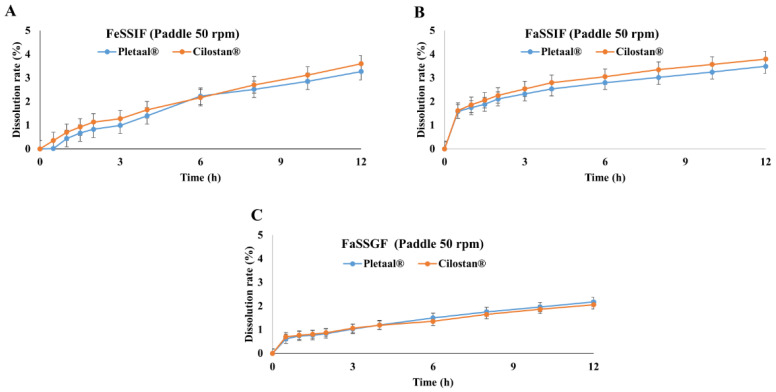
In vitro release profiles of Pletaal^®^ SR 200 mg capsules and Cilostan^®^ CR 200 mg tablets with a paddle method at 50 rpm. (**A**) Release medium FeSSIF with paddle at 50 rpm, (**B**) telease medium FaSSIF with paddle at 50 rpm, and (**C**) telease medium FaSSGF with paddle methods at 50 rpm.

Fed state simulated intestinal fluid (FeSSIF), fasted state simulated intestinal fluid (FaSSIF), and fasted state simulated gastric fluid (FaSSGF) were also used for release testing of extended-release dosage forms. Pletaal^®^ SR 200 mg capsules and Cilostan^®^ CR 200 mg tablets showed very low release levels (<5%) in FeSSIF, FaSSIF, or FaSSGF media (Figure 3A–C). Borelevant media (FaSSIF, FeSSIF, and FaSSGF) stimulate fluids to provide a more accurate prediction of in vivo behavior compared to simpler media. FaSSIF and FeSSIF contain bile salts and lecithin, which can form micelles. These micelles can solubilize lipophilic drugs, potentially reducing the free drug available for release. FaSSGF has a highly acidic pH to mimic the fasted state of the stomach. The drugs that are more soluble at neutral or basic pH showed low release. In these release media, a greater amount of capsules and tablets remained after completing the test compared with other testing media.

### 2.2. Quantitation of Cilostazol, OPC-13015, and OPC-13213 Using LC MS/MS

**Figure 4:**
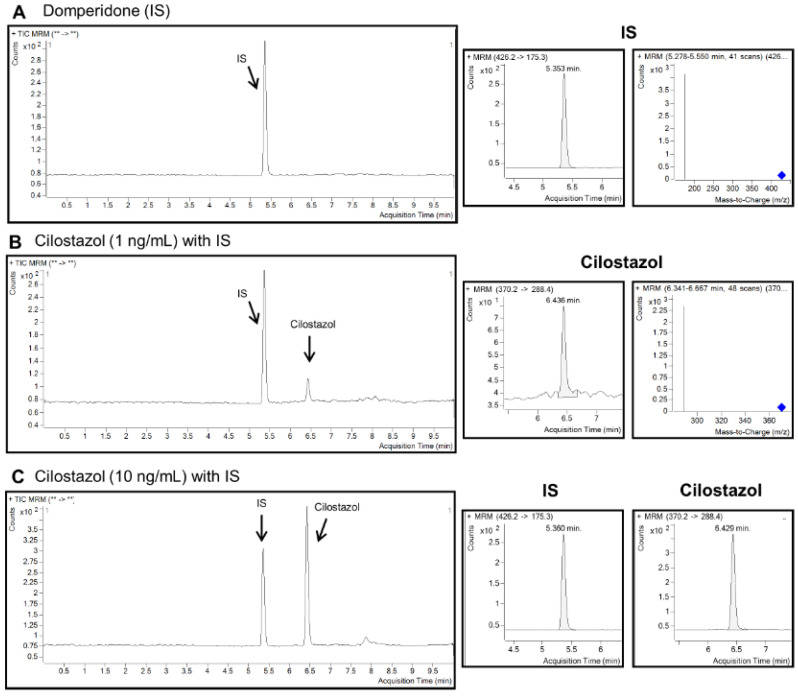

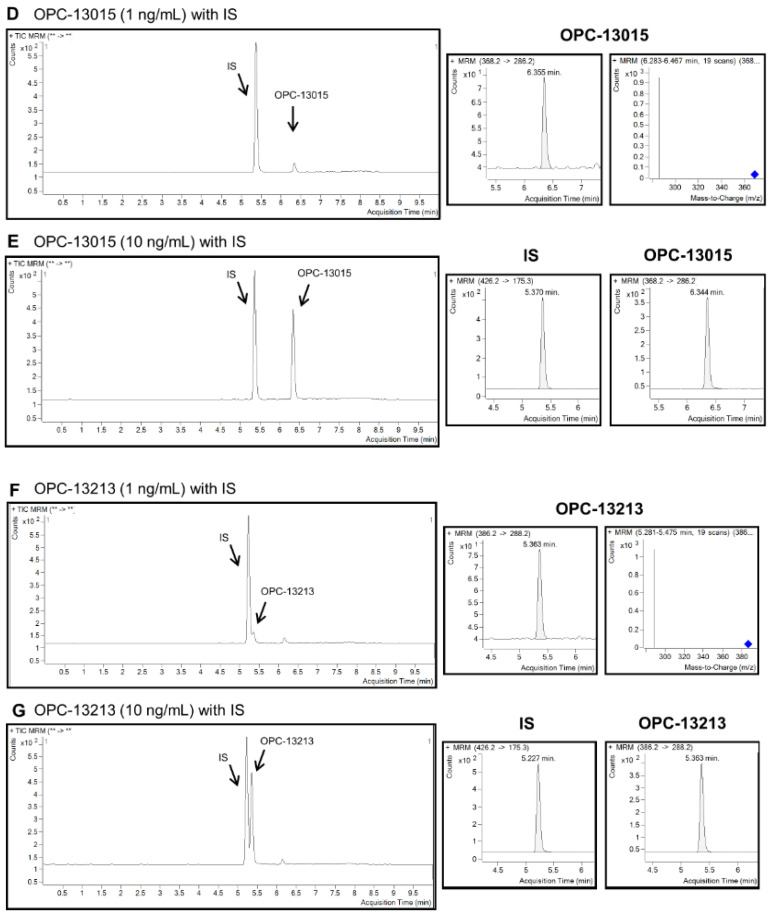
LC MS/MS chromatograms. (**A**) Domperidone (IS) internal standard at 1 ng/mL, (**B**) Cilostazol in blank beagle plasma spiked with known drug concentrations 1 ng/mL of Cilostazol along with IS 1 ng/mL of Domperidone, (**C**) Cilostazol in blank beagle plasma spiked with 10 ng/mL of Cilostazol along with 1 ng/mL of IS, (**D**) OPC-13015 in blank beagle plasma spiked with known drug concentrations 1 ng/mL of OPC-13015 along with IS 1 ng/mL of Domperidone, (**E**) OPC-13015 in blank beagle plasma spiked with 10 ng/mL of OPC-13015 along with 1 ng/mL of IS, (**F**) OPC-13212 in blank beagle plasma spiked with known drug concentrations 1 ng/mL of OPC-13212 along with IS 1 ng/mL of Domperidone, and (**G**) OPC-13212 in blank beagle plasma spiked with 10 ng/mL of OPC-13212 along with 1 ng/mL of IS. ** The transmission ion to a product ion.

The plasma concentrations of each compound type in plasma were quantified in both treatment groups (Pletaal^®^ SR 200 mg capsules and Cilostan^®^ CR 200 mg tablets) concerning the standard curve. The peak of Domperidone, an internal standard (IS), showed up earlier than the peaks of Cilostazol or metabolites (Figure 4A). The peak separation of IS and Cilostazol in the extracted blank plasma spiked with the Cilostazol standard reagent at the concentration of the lower limit of quantitation (LLOQ), 1 ng/mL of Cilostazol (Figure 4B). Peaks of IS and Cilostazol were well-separated in the extracted plasma spiked with 10 ng/mL of Cilostazol (Figure 4C). For five different batches of the extracted plasma spiked with 10 ng/mL of standard Cilostazol, OPC-13015, or OPC-13213, the retention time of Cilostazol, OPC-13015, and OPC-13213 was confirmed as 6.43, 6.34, and 5.36 min on average (n = 5), respectively. Inter-day and intra-day validation was also confirmed for Cilostazol, OPC-13015, and OPC-13213. Intra-day and inter-day RSD (%) were found to be lower than 15% (accuracy existed between 80 and 120%). Concerning the extraction efficiency from plasma, recovery (%) was verified for each compound type by comparing the peak response ratio of the extracted plasma and unextracted solution spiked with each standard of Cilostazol, OPC-13015, or OPC-13213. The recovery results showed that the extraction procedure used in this study was demonstrated as efficient for detecting concentrations of Cilostazol and its metabolites (OPC-13015 and OPC-13213) in plasma samples.

### 2.3. In Vivo PK Evaluation of Extended-Release Cilostazol Formulations under the Fasted and Fed Condition

**Figure 5:**
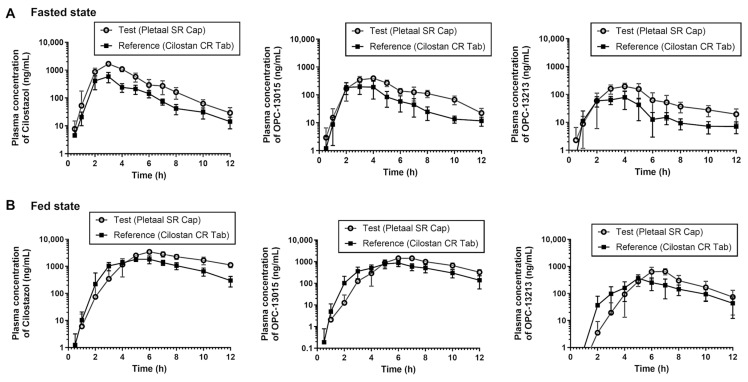
Plasma concentration vs. time profile of Cilostazol, OPC-13015, OPC-13213 in beagle dog plasma. (**A**) Fast and (**B**) feed conditions after a single oral administration of Pletaal^®^ SR 200 mg capsule or Cilostan^®^ CR 200 mg tablet.

Two Cilostazol oral extended-release formulations were analyzed for in vivo PK in beagle dogs under fasted or fed conditions. PK profiles in treatment groups of oral formulations under the fasted or fed condition were depicted using time vs. plasma concentrations of Cilostazol or its metabolites (OPC-13015 and OPC-13213) (Figure 5). As shown in Figure 5A, in the fasted condition, plasma concentrations of each compound type with oral administration of Pletaal^®^ SR 200 mg capsule was higher than that of oral administration of Cilostan^®^ CR 200 mg tablets. As shown in Table 1, under fasted state, the dogs were administered Pletaal^®^ SR 200 mg capsules and showed higher Cmax, AUC_0-t_, and AUC_0-inf_ for Cilostazol and metabolites (OPC-13015 and OPC-13213) than Cilostan^®^ CR 200 mg tablets. The geometric mean (GM) of maximal plasma concentration (Cmax) of Cilostazol in the Pletaal^®^ SR 200 mg capsules was 1656.83 ng/mL, CV (coefficient of variation) = 16.4%, whereas the GM of Cmax of Cilostazol in the Cilostan^®^ CR 200 mg tablets was 655.78 ng/mL, CV = 18.2%, (Table 2). The GM values of AUC_0-t_ and AUC_0-inf_ of Cilostazol in the Pletaal^®^ SR 200 mg capsules were 5203.01 h·ng/mL (5.9%) and 5282.45 h·ng/mL (6.2%), whereas in the Cilostan^®^ CR 200 mg tablets they were 1799.13 h·ng/mL (17.4%) and 1837.97 h·ng/mL (17.5%), respectively.

Beagle dogs were also evaluated for PK under the fed state with a cross-over design. Plasma concentrations of Cilostazol or metabolites in beagle dogs under the fed condition are shown in Figure 5B. The dog group administered Pletaal^®^ SR 200 mg capsules showed higher Cmax, AUC_0-t_, and AUC_0-inf_ for Cilostazol and its metabolites (OPC-13015 and OPC-13213) than that of Cilostan^®^ CR 200 mg tablet (Table 2). The GM of Cmax of Cilostazol in the Pletaal^®^ SR 200 mg capsule under the fed state was 3477.43 ng/mL, CV = 11.2%, whereas the GM of Cmax of Cilostazol in the Cilostan^®^ CR 200 mg tablet under the fed state was 2144.69 ng/mL, CV = 16.7%, *p* < 0.05 (Table 2). Based on the GM values, AUC_0-t_ and AUC_0-inf_ of Cilostazol in the Pletaal^®^ SR 200 mg capsule was 17,978.47 h·ng/mL (16.9%) and 24,445.11 h·ng/mL (17.1%), whereas in Cilostan^®^ CR 200 mg tablet it was 10,776.83 h·ng/mL (9.7%) and 11,966.19 h·ng/mL (9.9%), respectively. PK parameters (Cmax, AUC_0-t_, and AUC_0-inf_) for Cilostazol and metabolite (OPC-13015 and OPC-13213) between the Pletaal^®^ SR 200 mg capsule and Cilostan^®^ CR 200 mg tablet were significant (Table 2).

## 3. Discussion

The therapeutic effectiveness of dosage forms is a critical aspect of pharmaceutical science and patient care. It depends on a combination of formulation design, pharmacokinetic properties, patient factors, and treatment adherence regimens [23]. Formulation scientists continuously strive to develop innovative dosage forms that maximize drug efficacy, safety, and patient convenience [8,24,25]. Appropriate formulation design is an important parameter for pharmaceutical development that aims to ensure product quality by designing and controlling manufacturing processes [23,25]. It involves the application of scientific principles and risk-management strategies throughout the product lifecycle, from initial development to commercial production [26]. Cilostazol is a Biopharmaceutics Classification System (BCS) class II drug having poor aqueous solubility, slow oral absorption, and high permeability [27]. It is a neutral molecule with low water solubility (3.34 µg/mL) [28], and the permeability coefficient in the Caco-2 cell monolayer model was above 1 × 10^−6^ cm/s [29]. Generally, drugs with low aqueous solubility have a higher absorption rate when they are taken with a meal. The mechanisms behind higher absorption of Cilostazol after meal are increased biliary secretion, large volume of gastric fluid, change in gastric pH, and/or increased gastric emptying time [21,30]. Bramer et al. reported that after oral dosing of 100 mg Cilostazol tablet with a high-fat breakfast, both the Cmax and AUC of Cilostazol increased by 90% and 25%, respectively, compared with the fasted condition in humans [31].

In vitro, release results showed that Cilostan^®^ CR 200 mg tablet has 2 times higher release than that of Pletaal^®^ SR 200 mg capsule at release medium containing 0.5% SLS at pH 1.2. For the release medium containing 0.5% SLS at pH 4, both of the extended-release formulations showed less than 20% drug release. Similarly, for the release medium containing 0.5% SLS at pH 6.8, the Cilostan^®^ CR 200 mg tablet exhibits drug release 2.5 times higher than the Pletaal^®^ SR 200 mg capsule at 12 h. The slow release of drugs in acidic environments such as pH 1.2 and pH 4.0 enables enhanced bioavailability of certain drugs by facilitating their absorption in the gastrointestinal tract. This can be particularly beneficial for drugs with poor solubility or absorption characteristics. The release and diffusion rates of the drug within the delivery system can vary depending on the pH environment. Factors such as the thickness of the polymer matrix, drug–polymer interaction, and diffusion coefficients can all affect the release kinetics at different pH levels [32]. Therefore, the release from pH 4 seems to be slower than that from pH 1.2.

In pseudo gastric release media, Pletaal^®^ 200 mg capsules, with pH changes from 1.2 to 6.8 with 0.5% SLS, reached a higher release level of 60%, and with pH changes from 1.2 to 6.8 with 1% SLS, the release reached up to 68% at 12 h. Similarly, in the release profile for Cilostan^®^ CR 200 mg tablets at the same release medium condition, with pH changes from 1.2 to 6.8 with 0.5% SLS, the drug release was observed around 40% but in the case of pH changes from 1.2 to 6.8 with 1% SLS, the release profile for Cilostan^®^ CR 200 mg tablets decreased to 27% at 12 h. The surfactant content is increased when pseudo gastric release media are increased with a drug release profile of Pletaal^®^ 200 mg capsules.

PK studies with Cilostazol showed that fed effects or high-fat dietary effects might change drug absorption and clearance [31]; therefore, a once-daily instead of twice-daily dosing regimen was preferred. Considering various physicochemical and PK profiles and patient compliance with Cilostazol, sustained-release formulations are preferred to those of immediate-release formulations. Previously, PK studies of Cilostazol were focused on the comparison between immediate-release (IR) and sustained-release (SR) formulations or dosing regimens such as single or multiple dosing (i.e., twice-daily or once-daily dosing) [9,18]. Scientists also tested the equivalency of various dosage forms of Cilostazol (i.e., 2 × 50 mg vs. 1 × 100 mg or 2 × 100 mg vs. 1 × 200 mg) [18,31,33,34]. Cilostazol preclinical and clinical reports for food and high-fat diet showed slower oral absorption [31,33] than fast state. Rich fat meal or food intake time before drug administration also affected the PK profile and therapeutic efficacy of Cilostazol [27,31]. In summary, the food might critically influence the PK profiles of Cilostazol [21,35].

There are established LC MS/MS methods to analyze concentrations of Cilostazol, OPC-13015, and OPC-13213 in biological samples [36,37]. The specificity of Cilostazol or metabolites (OPC-13015 or OPC-13213) was confirmed with the appearance of a compound peak at the specific retention time (min). PK parameters (Cmax and AUC_0-inf_) were compared for fed and fasted states. Table 3 shows the ratio of geometric means of plasma PK parameters after oral administration of reference or test drug formulations in a fed/fasted state. In the fed state, after oral administration of the Pletaal^®^ SR 200 mg capsule, the Cmax and AUC_0-inf_ of Cilostazol increased by 2.10-fold and 4.63-fold with respect to the fasted state, respectively.

Miyake et al. reported that the time interval between food and drug administration can influence the PK parameters of sustained formulations of Cilostazol [27]. Their studies reported that PK parameters such as Cmax and AUC increased at 30 min, 1 h, 2 h, or 4 h after the food, compared with the fasted state. In the fed state, after oral administration of the Cilostan^®^ CR 200 mg tablet, the Cmax and AUC_0-inf_ of Cilostazol also increased by 3.27-fold and 6.51-fold, respectively, compared with the fasted state. Under the fasted state, the ratio of least squared geometric mean values (GMR), Cmax, AUC_0-t_, and AUC_0-inf_ of Cilostazol were 2.53-fold, 2.89-fold, and 2.87-fold higher in the Pletaal^®^ SR 200 mg capsule, compared with the Cilostan^®^ CR 200 mg tablet (Table 3). OPC-13015, the major active metabolite of Cilostazol, Cmax, AUC_0-t_, and AUC_0-inf_ were 1.54-fold, 2.14-fold, and 2.13-fold higher in the Pletaal^®^ SR 200 mg capsule compared with the ‘reference’ group. Moreover, OPC-13213 geometric means of Cmax, AUC_0-t_, and AUC_0-inf_ were 1.48-fold, 1.56-fold, and 1.67-fold higher in the group with the Pletaal^®^ SR 200 mg capsule, compared with the Cilostan^®^ CR 200 mg tablet.

The GMR values of Cilostazol, under the fed condition in the Pletaal^®^ SR 200 mg capsule showed 1.62-fold, 1.67-fold, and 2.04-fold higher values of Cmax, AUC_0-t_, and AUC_0-inf_, respectively, than that of the Cilostan^®^ CR 200 mg tablet (Table 4). Geometric means of Cmax, AUC_0-t_, and AUC_0-inf_ of OPC-13015 were 1.48-fold, 1.56-fold, and 1.67-fold higher in the group with the Pletaal^®^ SR 200 mg capsule, compared with the group with the Cilostan^®^ CR 200 mg tablet in the state of food intake. Similarly, in the fed condition, geometric mean values of Cmax, AUC_0-t_, and AUC_0-inf_ of OPC-13213 were 1.93-fold, 1.58-fold, and 1.55-fold higher in the group with the Pletaal^®^ SR 200 mg capsule, compared with the group with the Cilostan^®^ CR 200 mg tablet. The fasted state confirmed that these two oral formulations showed a lack of bioequivalence based on 0.8–1.25 of FDA guidance of the bioequivalence (90% CI for GMR).

In vitro, data showed that Pletaal^®^ SR 200 mg capsules have a much higher release rate than that of Cilostan^®^ CR 200 mg tablets in pseudo gastric release media. Similarly, an in vivo PK study showed that Pletaal^®^ SR 200 mg capsules showed higher C_max_ or AUC than Cilostan^®^ CR 200 mg tablets in both fasted and fed states. The phenomenon where there is a lower release of drug in vitro compared to a higher release in in vivo conditions can be attributed to several factors like pH variability, biological fluid components, absorption process, and drug-release mechanism. The lower and upper limit values of 90% CI for GMR suggested that these two extended-release formulations might not be bioequivalent according to the FDA criterion of bioequivalence. In vitro drug-release kinetics (Figure 2) and in vivo PK profiles (Figure 5) explain in vitro/in vivo profile correlation of two extended-release dosage forms. Also, in vitro and in vivo PK data suggested that these two formulations might not be equally effective in the body after oral administration under either fast or fed conditions. Our in vitro/in vivo results are consistent with a previously reported Cilostazol human study by Shin et al. [32].

## 4. Materials and Methods

### 4.1. Materials

Cilostazol (Table 5) and OPC-13015 (3,4-dehydrocilostazol, C_20_H_25_N_5_O_2_), and OPC-13213 (4′-trans-hydroxy-cilostazol, C_20_H_27_N_5_O_3_) were obtained from Otsuka Pharmaceutical Co., Ltd. (Tokushima, Japan). Internal standard Domperidone was purchased from Sigma-Aldrich (St. Louis, MO, USA). All chemicals were analytical grade and solvents for high-performance liquid chromatography (HPLC) and liquid chromatography/tandem mass spectroscopy (LC MS/MS) were of HPLC grade obtained from Honeywell Burdick & Jackson Labs (Morristown, NJ, USA).

### 4.2. In Vitro Release

Drug release profiles of two oral Cilostazol formulations were analyzed under physiological conditions using USP release apparatus type I (basket) and type II (paddle). Release medium compositions were tested including (1) pH 1.2 containing 0.5% SLS, (2) pH 4.0 containing 0.5% SLS, (3) pH 6.0 containing 0.5% SLS, (4) pH 6.8 containing 0.5% SLS, (5) FeSSIF, (6) FaSSIF, and (7) FaSSGF. In vitro drug-release studies for two oral formulations (Pletaal^®^ SR 200 mg capsule and Cilostan^®^ CR 200 mg tablet) were performed according to the US Pharmacopeia, using apparatus type II (Dissolution tester, PTWS-1220, PharmaTest Inc., Hainburg, Germany) filled with 900 mL of simulated stomach or intestinal fluid without pepsin (SIFsp). The medium was kept at 37 ± 0.5 °C and stirred at 50 rpm (sink conditions). The release testing was started after 30 min stirring with the addition of 4000 USP units of α-amylase enzyme in the vessel. In in vitro drug-release studies with a medium transfer, an apparatus type I method was applied. Each oral formulation (Pletaal^®^ SR 200 mg capsule or Cilostan^®^ CR 200 mg tablet) in the baskets was transferred from one to another medium: (1) medium at pH 1.2 containing 0.5% SLS to medium at pH 6.8 containing 0.5% SLS; (2) medium at pH 1.2 containing 1% SLS to medium at pH 6.8 containing 1% SLS; (3) FeSSGF to FaSSIF. The temperature was set at 37 ± 0.5 ºC with stirring at 100 rpm. The release testing was started after 30 min, starting with the addition of α-Amylase enzyme in the vessel and a sample solution from the vessel was taken out at each time point for further analysis. The sample solution (3 mL) was withdrawn at designed time points (0.5, 1, 1.5, 2, 3, 4, 6, 8, 10, and 12 h), and each aliquot was replaced with 3 mL of fresh release medium. Each sample solution was immediately analyzed by UV-Vis spectrophotometer (V730, JASCO Co., Tokyo, Japan) at 257 nm.

### 4.3. Animal

Male beagle dogs (9.5–11.5 kg) were used for the pharmacokinetic (PK) study. Beagle dogs were obtained from Orient Bio Inc. (Covance Research Products, Inc., Seongnam-si, Gyeonggi-Do, Republic of Korea) and acclimatized for 7 days with humane care in housing before performing the study. The animal study was conducted at the NDIC Co. Ltd. (Gwangju-si, Gyeonggi-Do, Republic of Korea). Animal care and in vivo experiments were performed by the international, national, and/or institutional guidelines provided by the Animal Experimentation Ethics Committee of NDIC Co., Ltd. following protocol no. P223008 (approval date: 30 April 2021). Dogs were housed under a controlled environment maintained at a temperature of 21 ± 2 °C, 150–300 Lux light, and 35–65% humidity. The dogs were fed with 300 g/day Purina Dow Chow (Argibrands Purina Korea Inc., Pyeongtaek-si, Gyeonggi-Do, Republic of Korea) during the designated time every afternoon during the acclimatization period.

### 4.4. Instrumentation: High-Performance Liquid Chromatography Coupled to Mass Spectrometry (LC MS/MS) 

Agilent 1260 series HPLC, equipped with a degasser, quaternary pump, and column oven, coupled to an Agilent 6460 Triple Quadrupole Mass detector with an electrospray ionization source (ESI) was used for the sample analysis. An Agilent Poroshell EC-C8 column, 3.0 × 50 mm, 2.7 µm, was used for the separation. Neta Scientific guard in-line filter (2 µL frits) was linked with the separation column. The column oven temperature was set at 30 °C and the temperature of the auto sampler was set at 10 °C. Mobile phase solution A (water with 0.1% formic acid) and mobile phase solution B (methanol:acetonitrile:water::90:2:8 with 0.1% formic acid) were used for analysis [36,37]. The solvent gradient method was applied for mobile phase solution A and mobile phase solution B at a flow rate of 0.3 mL/min through LC MS/MS. The following condition was maintained for the analysis: 100% of mobile phase solution A was maintained over 1 min and decreased to 40% of mobile phase solution A at 3 min, followed by reduction to 0% of mobile phase solution A and 100% of mobile phase solution B until 6 min, and finally it returned to 100% of mobile phase solution A at 7 min, and was maintained until 10 min for re-equilibration (sample analysis completed).

The instrument interface parameters were set as follows: drying gas temperature of 300 °C, gas flow of 10 L/min, nebulizer gas pressure of N_2_ of 45 psi, sheath gas temperature of 350 °C, sheath gas flow of 11 L/min, capillary voltage of 3500 V, and nozzle voltage of 500 V. The LC MS/MS was performed with a positive ion mode for the analysis of Cilostazol, OPC-13015, OPC-13213, and Domperidone. The optimum values of fragment voltages and collision energies (CEs), and the multiple reaction monitoring (MRM) scan modes were adopted to monitor transitions from the precursor to the product. There was an ion with mass-to-ion charge (m/z) ratio of Cilostazol (370.2 → 288.4; fragmentor 115 V; CE 15), OPC-13015 (368.2 → 286.2; fragmentor 135 V; CE 15), OPC-13213 (386.2 → 288.2; fragmentor 135 V; CE 15), and Domperidone (426.16 → 175.3; fragmentor 110 V; CE 30). MRM dwell time was set at 100 milliseconds. Chromatograms were captured with the Mass Hunter workstation software version B.05.00 (Agilent Technologies Inc., Santa Clara, CA, USA)

### 4.5. Standard Preparation and Method Validation

Stock solutions of Cilostazol, Domperidone, OPC-13015, and OPC-13213 were prepared in methanol (1 mg/mL). Further, each stock solution was diluted to 50 μg/mL with methanol followed by dilution to lower concentrations using mobile phase solution B solution (methanol:acetonitrile:water::90:2:8 with 0.1% formic acid). Working solutions were prepared by using the aliquots of each stock solution and blank beagle dog plasma. The calibration standard solutions of Cilostazol, OPC-13015, and OPC-13213 were prepared in a concentration range of 0, 0.5, 1, 2.5, 5, 10, 25, 50, 100, 250, 500, 1000, and 2000 ng/mL with the spiked Domperidone solution. Quality control (QC) solutions (0, 10, 100, 1000 ng/mL) were also prepared in the same manner as the calibration standard solutions. Beagle dog plasma samples were diluted with the help of calibration standard solution and QC solution for the further analysis process.

The linearity of calibration standard curves of Cilostazol, OPC-13015, and OPC-13213 was validated in the concentration range of 0–2000 ng/mL of each compound by using a linear regression method. Calibration curves were prepared based on data obtained from the standard solution using blank plasma. The peak area ratio was calculated by dividing the peak area of the compound by the peak area of Domperidone and calibration curves were depicted for the nominal compound concentration vs. peak area ratio obtained from each LC MS/MS chromatogram. Inter-day validation (between-day precision) was confirmed based on relative standard deviation (RSD) percentage based on calculations of peak area ratios for each concentration of Cilostazol, OPC-13015, and OPC-13213 for five samples (n = 5) using extracted calibration standard solutions by LC MS/MS on five consecutive days. For intra-day validation, the above procedure was followed but analysis was performed repeatedly. The accuracy of the LC MS/MS method and the acquired concentrations of Cilostazol, OPC-13015, and OPC-13213 from the calibration curves were compared with the nominal concentrations of each compound. Inter-day or intra-day precisions were acceptable within ±20% of the lower limit of quantitation and ±15% of the over limit of quantitation. Finally, the accuracy of the instrumental analysis was acceptable within 80–120% of the nominal concentrations of each compound. The plasma extraction efficiency was verified using the standard solutions of each compound type by comparing peak response ratios of the extracted solution and unextracted solution spiked with each standard reagent at the determined concentration of the compound.

### 4.6. Two-Period Crossover Study

First, for the fast state study, group 1 and group 2, beagle dogs were fasted for 16 h before the PK study, and they were orally administered each drug formulation (Pletaal^®^ SR 200 mg capsule for group 1 (n = 5) and Cilostan^®^ CR 200 mg tablet for group 2 (n = 5) with 20mL of water). One week was set for a wash-out period between period 1 and period 2 for each group. Group 1 (n = 5) was administered the Cilostan^®^ CR 200 mg tablet and group 2 (n = 5) was administered the Pletaal^®^ SR 200 mg capsule for a crossover PK study. A four-week wash-out period was set for each group after the blood samplings under the fasted state were finished.

In the fed-state PK study, beagle dogs in group 1 and group 2 were fasted for 16 h before the study, and then they were fed 200 mL (1.5 kcal/mL; 300 kcal composed of protein:fat:carbohydrate::17:30:53) of Carewell 1.5 plus liquid meal (Korea Enteral Foods, Co. Ltd., Seoul, Republic of Korea) 30 min before drug administration. Then, beagle dogs in group 1 (n = 5) and group 2 (n = 5) were orally administered a Pletaal^®^ SR 200 mg capsule and Cilostan^®^ CR CR 200 mg tablet with 20 mL of water, respectively. One week was set for a wash-out period between the experiments. As a cross-over PK study, the beagle dogs in group 1(n = 5) were administered a Cilostan^®^ CR 200 mg tablet, and those in group 2 (n = 5) were administered a Pletaal^®^ SR 200 mg capsule with water after 30 min of 200 mL of Carewell 1.5 plus liquid meal (1.5 kcal/mL; 300 kcal composed of protein:fat:carbohydrate::17:30:53). In each fasted or fed-state group study, blood samples were drawn after oral drug administration from the jugular vein into the heparin-coating vacutainer tubes at 0 h (pre-dose), 30 min, 1 h, 2 h, 3 h, 4 h, 5 h, 6 h, 7 h, 8 h, 10 h, and 12 h. Blood samples were centrifuged at 4000 rpm for 10 min at 4 °C to obtain plasma samples. Plasma was stored at −70 °C for the quantitation of Cilostazol, OPC-13015, and OPC-13213 by LC MS/MS.

### 4.7. Analysis of Cilostazol, OPC-13015, and OPC-13213

Plasma extraction was carried out to measure concentrations of Cilostazol or its metabolite (OPC-13015 or OPC-13213) after administration of the Cilostazol-containing extended-release formulation (Pletaal^®^ SR 200 mg capsule and Cilostan^®^ CR 200 mg tablet). A 400 μL amount of plasma sample, 80 μL of cold acetonitrile, and 40 μL of Domperidone (5 ng/mL) solution were mixed on a vortex for 1 min followed by addition of 200 mL of 1 mM sodium carbonate (Na_2_CO_3_) again vortexed for 1 min. Then, 2.4 mL of methyl *tert*-butyl ether was added and mixed on a vortex for 3 min. The final solution was centrifuged at 13,000 rpm for 10 min and 1.6 mL aliquot of organic supernatant was transferred to the tube for evaporation using the centrifugal evaporator (EYELA CVE-2200, Tokyo, Japan) with 1400 rpm vacuum speed. After drying, the residue in the tube was reconstituted in 200 mL of mobile phase solution B by vortexing. Samples were filtered using a syringe filter (0.22 μm PVDF), and 5 μL of filtered solution was kept in the autosampler vial for LC MS/MS analysis. Calibration curves prepared from standard reagents of Cilostazol or metabolites (OPC-13015, and OPC-13213) were used to calculate concentrations of each compound present in plasma. Plots of plasma concentrations versus time were depicted for oral administration of Pletaal^®^ SR 200 mg capsule vs. Cilostan^®^ CR 200 mg tablet.

### 4.8. PK Analysis

In vivo, PK data were analyzed using the Phoenix WinNonLin program (version 8.3; Certara Co., Princeton, NJ, USA). The maximum plasma concentration (Cmax), and the area under the concentration–time curve from time zero to the last measured time (AUC_0-t_) or infinity (AUC_0-inf_) were calculated using the non-compartmental analysis (NCA) method. All data in tables were expressed as mean ± standard deviations (SDs).

### 4.9. Statistical Analysis

Statistical analysis was performed using GraphPad Prism 9.5.1 and *p*-value < 0.05 was evaluated as significant. PK parameters obtained from the experiments under the fed condition and the fast condition were also subject to statistical analysis. Bioequivalence was assessed by 90% confidence intervals (CIs) for the geometric mean ratio of Pletaal^®^ SR 200 mg capsule and Cilostan^®^ CR 200 mg tablet, respectively. The Pletaal^®^ SR 200 mg capsule and Cilostan^®^ CR 200 mg tablet oral formulations were regarded as bioequivalent if 90% confidence limits existed between 0.80 and 1.25.

## 5. Conclusions

Two commercial oral extended-release formulations of Cilostazol (Pletaal^®^ SR 200 mg capsule and Cilostan^®^ CR 200 mg tablet) were compared for in vitro drug release kinetics and in vivo PK profiles obtained from beagle dogs. In vitro, drug release profiles under physiological mimicked conditions showed that Pletaal^®^ SR 200 mg capsule has better release than that of Cilostan^®^ CR 200 mg tablet after oral administration. In vivo PK data showed that the Pletaal^®^ SR 200 mg capsule exhibited higher AUC and C_max_ than the Cilostan^®^ CR 200 mg tablet under both fast and fed conditions which is similar to previously reported clinical bioequivalence tests in healthy Korean volunteers. In vitro and in vivo PK correlation suggested that these two formulations might not be equally effective in the body after oral administration under either fast or fed conditions. These study results showed that Pletaal^®^ SR 200 mg capsules might not be interchangeable with Cilostan^®^ CR 200 mg tablets.

## Figures and Tables

**Table 1:** In vivo PK parameters of Cilostazol and metabolites (OPC-13015 and OPC-13213) in beagle dog plasma after Pletaal^®^ SR 200 mg capsule and Cilostan^®^ CR 200 mg tablet oral administration under fast state.

Compound Type	PK Parameters under Fast State	Geometric Mean ± SD (CV, %)		Ratio (Pletaal^®^ SR 200 mg Capsule/Cilostan^®^ CR 200 mg Tablet)	*p*-Value
		Pletaal^®^ SR 200 mg Capsule	Cilostan^®^ CR 200 mg Tablet		
Cilostazol	Cmax (ng/mL)	1656.83 ± 1.17 (16.4%)	655.78 ± 1.19 (18.2%)	2.53	<0.001
	AUC_0-t_ (h·ng/mL)	5203.01 ± 1.06 (5.9%)	1799.13 ± 1.19 (17.4%)	2.89	<0.001
	AUC_0-inf_ (h·ng/mL)	5282.45 ± 1.07 (6.2%)	1837.97 ± 1.19 (17.5%)	2.87	<0.001
OPC-13015	Cmax (ng/mL)	427.75 ± 1.17 (15.5%)	277.25 ± 1.34 (25.7%)	1.54	<0.001
	AUC_0-t_ (h·ng/mL)	1769.29 ± 1.12 (12.3%)	826.05 ± 1.23 (20.0%)	2.14	<0.001
	AUC_0-inf_ (h·ng/mL)	1853.58 ± 1.13 (13.9%)	872.19 ± 1.23 (19.5%)	2.12	<0.001
OPC-13213	Cmax (ng/mL)	241.29 ± 1.11 (10.0%)	95.51 ± 1.35 (28.1%)	2.52	<0.001
	AUC_0-t_ (h·ng/mL)	817.66 ± 1.10 (9.7%)	302.80 ± 1.37 (29.1%)	2.70	<0.001
	AUC_0-inf_ (h·ng/mL)	937.21 ± 1.19 (17.9%)	351.70 ± 1.30 (26.5%)	2.66	<0.001

C_max_ = maximum plasma concentration of each compound; AUC_0-t_ = AUC from zero to the measured last time point; AUC_0-inf_ = AUC from zero to infinity.

**Table 2:** In vivo PK parameters of Cilostazol and metabolites (OPC-13015 and OPC-13213) in beagle dog plasma after Pletaal^®^ SR 200 mg capsule and Cilostan^®^ CR 200 mg tablet oral administration under fed state.

Compound Type	PK Parameters under Fed State	Geometric Mean ± SD (CV, %)		Ratio (Pletaal^®^ SR 200 mg Capsule/Cilostan^®^ CR 200 mg Tablet)	*p*-Value
		Pletaal^®^ SR 200 mg Capsule	Cilostan^®^ CR 200 mg Tablet		
Cilostazol	Cmax (ng/mL)	3477.43 ± 1.13 (11.2%)	2144.69 ± 1.17 (16.7%)	1.62	<0.001
	AUC_0-t_ (h·ng/mL)	17,978.47 ± 1.19 (16.9%)	10,776.83 ± 1.11 (9.7%)	1.67	<0.001
	AUC_0-inf_ (h·ng/mL)	24,445.11 ± 1.18 (17.1%)	11,966.19 ± 1.10 (9.9%)	2.04	<0.001
OPC-13015	Cmax (ng/mL)	1557.11 ± 1.17 (15.7%)	1054.97 ± 1.22 (18.2%)	1.48	<0.001
	AUC_0-t_ (h·ng/mL)	7298.10 ± 1.11 (10.4%)	4679.33 ± 1.18 (15.8%)	1.56	<0.001
	AUC_0-inf_ (h·ng/mL)	8607.24 ± 1.17 (15.8%)	5161.49 ± 1.22 (19.5%)	1.67	<0.001
OPC-13213	Cmax (ng/mL)	794.39 ± 1.22 (17.9%)	412.49 ± 1.19 (15.3%)	1.93	<0.001
	AUC_0-t_ (h·ng/mL)	2445.29 ± 1.34 (27.9%)	1544.16 ± 1.18 (16.8%)	1.58	<0.001
	AUC_0-inf_ (h·ng/mL)	2687.73 ± 1.36 (30.7%)	1732.12 ± 1.17 (15.8%)	1.55	<0.001

C_max_ = maximum plasma concentration of each compound; AUC_0-t_ = AUC from zero to the measured last time point; AUC_0-inf_ = AUC from zero to infinity.

**Table 3:** In vivo estimation of food effect based on ratios of geometric mean values of Cilostazol and metabolites (OPC-13015 and OPC-13213) for Pletaal^®^ SR 200 mg capsule and Cilostan^®^ CR 200 mg tablet in fed/fasted state.

Compound Type	PK Parameters under Fed State	Fed/Fast Geometric Mean Ratio	
		Pletaal^®^ SR 200 mg Capsule	Cilostan^®^ CR 200 mg Tablet
Cilostazol	Cmax (ng/mL)	2.10	3.27
	AUC_0-inf_ (h·ng/mL)	4.63	6.51
OPC-13015	Cmax (ng/mL)	3.64	3.81
	AUC_0-inf_ (h·ng/mL)	4.64	5.92
OPC-13213	Cmax (ng/mL)	3.29	4.32
	AUC_0-inf_ (h·ng/mL)	2.87	4.92

**Table 4:** In vivo PK parameters and geometric mean ratio (GMR) of Cilostazol, OPC-13015, and OPC-13213 after oral administration of Pletaal^®^ SR 200 mg capsule or Cilostan^®^ CR 200 mg tablet in beagle dogs under the fast or fed condition.

Compound Type		PK Parameters	Geometric Mean Ratio (GMR) ^a^ (Pletaal^®^ SR 200 mg Capsule/Cilostan^®^ CR 200 mg Tablet)	90% of CI for GMR ^b^ (Lower Limit–Upper Limit)
Cilostazol	Under fasted state	Cmax (ng/mL)	1.6214	2.1396–2.9833
		AUC_0-t_ (h·ng/mL)	1.6683	2.5665–3.2587
		AUC_0-inf_ (h·ng/mL)	2.0428	2.5568–3.2307
	Under fed state	Cmax (ng/mL)	1.6214	1.4126–1.8611
		AUC_0-t_ (h·ng/mL)	1.6683	1.4242–1.9542
		AUC_0-inf_ (h·ng/mL)	2.0428	1.7497–2.3851
OPC-13015	Under fasted state	Cmax (ng/mL)	1.5428	1.3798–1.7251
		AUC_0-t_ (h·ng/mL)	2.1419	1.8130–2.5304
		AUC_0-inf_ (h·ng/mL)	2.1252	1.7915–2.5210
	Under fed state	Cmax (ng/mL)	1.4760	1.2364–1.7619
		AUC_0-t_ (h·ng/mL)	1.5596	1.4344–1.6959
		AUC_0-inf_ (h·ng/mL)	1.6676	1.5352–1.8114
OPC-13213	Under fasted state	Cmax (ng/mL)	1.4760	1.2364–1.7619
		AUC_0-t_ (h·ng/mL)	1.5596	1.4344–1.6959
		AUC_0-inf_ (h·ng/mL)	1.6676	1.5352–1.8114
	Under fed state	Cmax (ng/mL)	1.9258	1.6157–2.2956
		AUC_0-t_ (h·ng/mL)	1.5836	1.2794–1.9600
		AUC_0-inf_ (h·ng/mL)	1.5517	1.2862–1.8720

**^a^** % ratio of least-square geometric means (LSM); **^b^** 90% confidence interval (CI) of the ratio.

**Table 5:** Two extended-release Cilostazol product details.

Product	Batch Number	Shelf-Life	Expiry Date	Ingredients
Pletaal^®^ SR 200 mg capsule	PC215008B	36 Months	11 May 2024	Cilostazol, Microcrystalline cellulose, Citric acid, Corn starch, pregelatinized starch, Polysorbate 80, Silicon dioxide, Hypromellose capsule
	PC215009B			
	PC215010B			
Cilostan^®^ CR 200 mg tablet	6125105		3 January 2024	Cilostazol, Microcrystalline cellulose, Carbomer, Povidone, Magnesium stearate, Sodium Lauryl Sulfate, Silicon dioxide, Hypromellose
	6125106		15 February 2024	
	6125113		5 April 2024	

## Data Availability

All the data are available within this article.
